# Supplementary material for: Attachment and epistemic trust in junior and senior university students: The mediating role of affect regulation and mentalizing. Who is at risk?
Source: PLoS One. 2025 Aug 13;20(8):e0304749. doi: 10.1371/journal.pone.0304749 (PMC12349075; doi:10.1371/journal.pone.0304749)
Supplement: Attachment — Submitted filename: Response to Reviewers.docx [file pone.0304749.s001.docx]

Dear Editor and Reviewer, thank you for allowing us to revise and resubmit our manuscript and for your insightful comments and instructions to improve our manuscript. Please find our answers per comment as follows:

**-Reviewer’s Comment:** The manuscript makes a valuable contribution to the existing literature, particularly in the context of students' mental health, emotion regulation, and academic learning and achievement. However, several recommendations should be taken into consideration.

The title should be clearer, shorter, and more understandable. The three dots are not helpful in achieving clarity and are not in line with academic writing style.

**Answer**: We changed the title leaving out the dots and making it shorter

Attachment and epistemic trust in junior and senior university students. The mediating role of affect regulation and mentalizing. Who is at risk?

**-Reviewer’s Comment:** The abstract should provide a clearer explanation of the study's rationale, highlighting its relevance for student populations and briefly explaining its implications.

**Answer:** We made the following alterations to the Abstract to make it more readable.

Research on emotional factors and mental health in higher education has gained traction. Much attention has focused on first-year students as a potentially at-risk group, though some studies suggest that all students might face similar risks. This study examines differences between junior and senior undergraduates in terms of mentalizing, emotion regulation (ER), and psychological mindedness, involving cognitive capacities significantly developed by late adolescence. These constructs relate to understanding one's own and others' mental states, potentially mediating the relationship between attachment and epistemic trust (ET). The current study includes 460 undergraduate students, most of whom are female (96%). Results show that senior students score higher on reappraisal, certainty, and interest/curiosity compared to junior students. However, these factors did not mediate the relationship between anxious attachment orientation and ET. Certainty and interest/curiosity mediated the relationship between avoidant attachment orientation and ET, suggesting similar mediation patterns for junior and senior students. On the other hand, suppression and uncertainty/confusion were critical mediators in the relationship between insecure (anxious and avoidant) attachment orientations and epistemic trust. Findings suggest that universities should (a) foster environments that support psychological capacity and facilitate positive learning experiences, and (b) enhance epistemic trust through safe curiosity and develop protective and preventive interventions.

**-Reviewer’s Comment:** The introduction is repetitive; please shorten the sections focusing on the main hypothesis, avoid redundancy in the hypothesis, and clearly define the variables based on the instruments used.

**Answer:** This point has been remedied accordingly (p. 10).

*Based on the Desatnik et al. study (2023), it is hypothesized that senior students will show higher levels of emotion regulation, psychological mindedness, and mentalizing compared to junior students (H1). Furthermore, junior students are expected to report the negative aspects of mentalizing (uncertainty/confusion) to mediate the relationship between insecure attachment and ET (H2), and senior students will report the positive aspects of mentalizing (certainty, interest/curiosity) to mediate the relationship between insecure attachment and ET (H3). Suppression is expected to mediate the relationship between avoidance attachment and ET for both junior and senior students (H4); reappraisal is hypothesized to mediate the relationship between anxious attachment and ET only for senior students. Suppression will mediate the relationship between anxious attachment and epistemic trust reported only by senior students (H5).*

**-Reviewer’s Comment:** Provide more details on the response rate. Provide information if there were any exclusion criteria. The sample has a gender imbalance, and this affects the generalizability of findings.

Did the authors consider steps to reduce gender imbalance and recruit more male students?

**Answer:** The gender imbalance in the sample is expected since students from social and education sciences were mostly recruited (p.11). This limitation is mentioned in the Discussion section.

**-Reviewer’s Comment:** I recommend detailing which social media platforms were used.

**Answer:** We added a list of social media platforms we used (p. 11).

**-Reviewer’s Comment:** Why did you choose ECR-R as a measurement? It does not measure secure attachment. How could the sample be categorized into avoidant or anxious attachment styles? It is not logical to divide the sample into these two categories. How did you code the instrument? Please clarify and state how ECR-R measures attachment dimensions, and how secure attachment is not measured directly but is inferred by low scores on avoidant and anxious attachment scores. Put this in the limitations of the study.

**Answer:** The instrument chosen is the one most widely used to assess adult attachment. It is an instrument with good psychometric properties (Wei et al., 2007). It is indicative that the original article testing the psychometric properties of the short form of the scale has been cited by 1821 documents. The scale has been adapted into many languages with sound psychometric properties (e.g., Brenk-Franz et al., 2018; Imran et al., 2020). Adult attachment is predominately conceptualized as a dimensional construct since attachment security and attachment anxiety and avoidance (insecurity) exist along a continuum (Fraley et al., 2015). More details on the coding of the instrument have been added in the methods section (p. 12) and the limitations of using a dimensional measure have been mentioned in the discussion section (p. 28).

**-Reviewer’s Comment:** Please give information on the validity of the instruments and specifically state the reliability of the instruments from the sample compared to that of the original scales.

**Answer:** Further information about construct validity and original scales’ reliability was added to the text (p. 14-15). Specifically, all data are presented in a table (Table 3) so the reader can have a clear vision inspection of the relevant information.

**-Reviewer’s Comment:** It is not usual to have junior and senior students at 72 years old. Please explain that. If the sample is part of a broader community, please explain.

**Answer:** The case is exactly as mentioned in the comment. We proceeded to clarify this in the text by omitting redundant information and focusing on the present study (p.11).

**-Reviewer’s Comment:** Please follow the journal guidelines for formatting tables.

**Answer:** The required corrections were made according to the journal’s guidelines.

**-Reviewer’s Comment:** Briefly explain why the bootstrap method is used and briefly explain Zhao et al.'s approach for interpreting the mediation model.

**Answer:** We provided a brief explanation in the text for both procedures, bootstrapping and the interpretation of mediation models by Zhao et al. (2010).

**-Reviewer’s Comment:** Please divide the result section into subsections, such as descriptive and correlations analysis, and mediation analyses, as it is difficult to read.

**Answer:** We changed the titles of the subsections and this allowed us to re-arrange the text mostly of the assumptions section, making it more consistent and readable (p.17-18).

**-Reviewer’s Comment:** How is categorizing the correlations as mild, moderate, and large helpful for your hypothesis? Explain why you categorized the correlations and provide correlation coefficients in the writing of the results.

**Answer:** The sections of the data analyses concerning the correlations (p.14) and the findings (p. 15-17) were revisited to remedy this comment. We changed the nomenclature slightly to follow Guildford’s classification and added the appropriate references. Furthermore, the correlation coefficients were added to the text, making it easier for the reader to follow the results in Table 2.

**-Reviewer’s Comment:** In the result section, please just state the findings but do not interpret them.

**Answer:** We made some adjustments to omit sentences that could seem to explain the results.

**-Reviewer’s Comment:** The discussion section is long and ideas are repetitive. It is difficult to read. Elaborate more on findings that are not consistent with the literature.

**Answer:** We made some adjustments to omit sentences that were repetitive and make the text more concise. The excerpts that were crossed out can be seen in the Manuscript with Track Changes, especially in the beginning of the Discussion (p. 22) and in the Implementation section (p. 28).
