# Supplementary material for: Attachment and epistemic trust in junior and senior university students: The mediating role of affect regulation and mentalizing. Who is at risk?
Source: PLoS One. 2025 Aug 13;20(8):e0304749. doi: 10.1371/journal.pone.0304749 (PMC12349075; doi:10.1371/journal.pone.0304749)
Supplement: Attachment — Submitted filename: Response to Reviewers 2.docx [file pone.0304749.s002.docx]

Dear Editor and Reviewer, thank you for your kind remarks on our manuscript and for your insightful comments and instructions to improve our manuscript. Please find our answers per comment as follows:

**-Reviewer’s Comment:** First, the authors report that the validity of the scales has been discussed elsewhere; however, no other information was provided. Simply stating that the validity of the scales has been discussed elsewhere is not transparent and does not provide at least a short description of the main report on the validity of the scales used in a different socio-cultural context.

*Answer:* The sample of the present sample (students) stems from a larger community sample. Therefore, the measures used are the same and they have been reported in detail as to their psychometric properties in Karagiannopoulou et al. (2024). For clarity, we added the appropriate statements in each of the measures in the relevant section (pp.12-13) and a more specific sentence about convergent and structural validity in the Results section (p.14).

**-Reviewer’s Comment:** Second, the authors use PROCESS for the mediation analysis; however, the program has serious limitations compared to other programs such as MPlus. The rationale for using PROCESS instead of other programs should be included, and the limitations section should address this while recommending more advanced and rigorous models for future studies.

*Answer:* This comment holds merit and resonates the discussion about the advantages and disadvantages of the statistical procedures used to estimate mediations/moderations. Concerning the use of PROCESS, it has been widely used in mediational analyses as being relatively easy and straight-forward to use, especially for simple mediations as is the case of our study. Furthermore, the use of PROCESS with bootstrapping compared to the Sobel test allows for “re-sampling while requiring fewer assumptions, provides a higher study power, and lowers the risk of falsely rejecting the null hypothesis” (Abu-Bader & Jones, 2021, p.57). Furthermore, Hayes et al. (2017) argue that factor-based SEM methods are better performing using PROCESS compared with other programs such as AMOS, LISREL, EQS, and Mplus, especially when it comes to the generation of interaction terms or sample size requirements. Our analyses are performed with observed variables, and although for more composite models Partial Least Square-SEM seems more appropriate, PROCESS is more suitable for singular models running ordinary least squares regressions (Sarstedt et al., 2020). A relevant suggestion for future complex mediation analyses was added in the Limitations section.

**-Reviewer’s Comment:** Third, in the discussion section, the authors overgeneralize their findings by interpreting the results using brain maturation concepts, despite the fact that no neural processes were measured. These interpretations are speculative, and the authors should be cautious, primarily recommending further studies to measure neurological mechanisms in combination with self-report data.

*Answer:* In the Discussion it is added that neuroimaging studies have also reported a discrepancy between suppression and reappraisal, beyond the self-report studies (p. 27). Furthermore, in the Limitations section it is suggested that “Including additional constructs could lead to a comprehensive model that integrates cognitive, emotional, and mental health aspects, enhancing our understanding of educational studies” (p.29).

**References:**

Abu-Bader, S., & Jones, T. V. (2021). Statistical mediation analysis using the sobel test and hayes SPSS process macro. *International Journal of Quantitative and Qualitative Research Methods*, *9*(1), 42-61.

Hayes, A. F., Montoya, A. K., & Rockwood, N. J. (2017). The analysis of mechanisms and their contingencies: PROCESS versus structural equation modeling. *Australasian Marketing Journal, 25*, 76–81.

Karagiannopoulou E, Milienos FS, Desatnik A, Rentzios C, Athanasopoulos V, Fonagy P. A short version of the reflective functioning questionnaire: Validation in a greek sample. *PloS One*. 2024; 19(2):e0298023. doi.org/10.1371/journal.pone.0298023

Sarstedt, M., Hair, J. F., Nitzl, C., Ringle, C. M., & Howard, M. C. (2020). Beyond a tandem analysis of SEM and PROCESS: Use of PLS-SEM for mediation analyses! *International Journal of Market Research, 62*(3), 288-299. <https://doi.org/10.1177/1470785320915686>
