# Supplementary material for: Attachment and epistemic trust in junior and senior university students: The mediating role of affect regulation and mentalizing. Who is at risk?
Source: PLoS One. 2025 Aug 13;20(8):e0304749. doi: 10.1371/journal.pone.0304749 (PMC12349075; doi:10.1371/journal.pone.0304749)
Supplement: Attachment — Submitted filename: Response to Reviewers 3.docx [file pone.0304749.s003.docx]

Dear Reviewers, we greatly appreciate the time and effort you have invested in reviewing our submission entitled “Attachment and epistemic trust in junior and senior university students. The mediating role of affect regulation and mentalizing. Who is at risk?”. We would like to thank you for your thoughtful and constructive comments on our manuscript. We have carefully considered all the comments and have revised the manuscript accordingly. Below you can find detailed responses to each comment:

Reviewer #1: I thank the authors and acknowledge their efforts to improve their manuscript. However, there are some further clarification that the authors should improve.

*-Comment:* First, the statement that PROCESS could better perform than MPLUS or SEM is misleading. PROCESS is effective in regression mediation analysis with observed variables but not with latent variables. Thus, PROCESS does not account for measurement error, does not provide a model fit in order to increase the possibility for generalizability and robustness of findings. This study uses self-report for psychological constructs, which are manifested as latent constructs, so claiming that is more suitable for observed variables is a key assumption violation. The literature that the authors provide is more focused on consumer behavior using observed metrics but not typically involving latent traits such as emotion regulation, mentalizing or attachment patterns. Please clarify this in the manuscript to be as clear as possible, in order for future studies to replicate with more advanced statistical models. Please review the citation in the paragraph of methodology: “Mediation hypotheses were tested using the PROCESS Procedure for SPSS…. (36 is not in line the citation with the reference) and then…. Analyses were performed with observed variables (not correct these are latent variables).

*Answer:* We thank the reviewer for this insightful and important comment. We acknowledge that our original statement may have been misleading in its comparison between PROCESS and more advanced SEM tools such as Mplus. As correctly pointed out, PROCESS is limited to mediation analyses using observed variables and does not account for measurement error, nor does it provide overall model fit indices. Given that our study involves self-reported measures of psychological constructs, which are best conceptualized as latent variables (e.g., emotion regulation, mentalizing, and attachment), the use of PROCESS indeed comes with methodological limitations. We have revised the manuscript accordingly to clarify that PROCESS was used as a pragmatic choice for estimating indirect effects with observed composite scores, but that this approach does not model latent variables or account for measurement error. We now explicitly state that this limits the generalizability and robustness of our findings and that future research should ideally replicate these findings using latent variable modeling via SEM approaches. Additionally, we have corrected the misstatement regarding "analyses being performed on observed variables" and have revised the language to reflect that composite scores were used as proxies for latent constructs. The incorrect citation [36] in the methodology section has also been corrected and now aligns with the reference list [63]. Finally, we omitted the source that previously corresponded to [89] and replaced it with a more suitable that describes the PROCESS approach by Hayes.

“…analytical framework to evaluate both direct and indirect effects with observed composite scores as proxies for latent variables [63]. All outcome variables were continuous, and the standard assumptions of ordinary least squares regression (normality, independence, and homoscedasticity) were tested [89].”

“Although PROCESS was used as a pragmatic choice for estimating indirect effects with observed composite scores, this approach does not model latent variables or account for measurement error, limiting the generalizability and robustness of the findings. In the future more complex models need to be examined using composite-based SEM methods such as partial least squares (PLS-SEM). ”

-*Comment:* Second, the brain and neuroimaging literature does not specifically address the current study aims, as this study does not include any brain or neural data. Thus, referring to brain maturation processes to explain self-report results is speculative and go beyond the focus of your data. Please, revise each paragraph that have been addressing brain and neural interpretation to your data and remove them. Please, explicitly acknowledge that your interpretations based on neural development are hypothetical and require empirical validation in future research that directly measures neurobiological process, please use the information as a tentative explanation but that is unmeasured in the current study and that it should be interpreted with cautiousness. Even if these studies provide a theoretical rationale for grouping of students, however they should not be used to interpret the results of the study directly. Furthermore, referring to brain maturation process in the discussion section risks overstating the evidential basis of your conclusions, as the study does not support these results empirically. Be explicit in the statement that studies of brain and neural development cannot test these claims directly in your study, and in the discussion section state that this interpretations are speculative and that future studies could test empirically, and please in the limitations state clearly that the lack of brain and neural findings limits the conclusions about underlying brain or neural mechanisms, please remove terms like mentalizing system activation as this term implies neural activation systems which were not measured in the current study. Focus more on behavioral or psychological terms like mentalizing processes.

*Answer:* In the text all neurological terminology was removed or replaced with phrases that imply psychological processes (instead of systems). We also added that interpretations tapping into neurological research were not based on the study’s findings and need to be further empirically tested. Please find below the respectful excerpts:

“From this perspective, mentalizing abilities and attachment relationships are seen as loosely interconnected [18; 19]. Moreover, empirical evidence strongly supports the idea that both the attachment and mentalizing processes play central roles in stress and emotion regulation [20].”

“Research suggests that mentalizing tends to improve with age, with evidence showing changes throughout adolescence and into early adulthood. Particularly, Desatnik et al., (2023) suggest that mentalizing ability increases gradually from early adolescence to young adulthood and keeps mature beyond it, aligning with brain development. Notably, females aged 17-18 showed different mentalizing patterns compared to those over 20 [8]. In this line of thinking, Poznyak et al., (2025) demonstrate that while basic mentalizing processes exist in adolescence, as people get older, they become more flexible in how they shift between the “self-other” representations [90].”

“Despite growing interest in the relationship between attachment and student learning and adjustment, research on the way mentalizing operates remains limited.”

“These changes are part of the broader emotional development process; studies show that the cognitive control aspects of reappraisal become more active with age [44].”

“In the context of these processes, trust in others as sources of social information fosters resilience through a salutogenic process, allowing individuals to gain from the stream of relevant information in the social environment [20].”

“Our perspective that exploring the attachment and mentalizing processes can inform understandings of learning, success, and adjustment in higher education aligns with Moreau et al.'s [13] caution against "overstating the environmental factors in success" compared to individual traits.”

“For junior students, interest/curiosity reduced the positive association between attachment avoidance and mistrust. This "beneficial" mediation may reflect developmental limitations that could be cautiously – given the self-report methodology of the present study – linked to research showing that brain regions responsible for understanding mental states remain immature until late adolescence [17].”

“Moreover, excessive certainty enhanced the negative relationship between avoidant attachment and epistemic trust reported by seniors, possibly suggesting that the development of more complex “self-other” processes might raise stress levels and activate negative schemata regarding others, thus triggering defensive independence. Therefore, cognitive maturation possibly inhibits controlled mentalizing [17; 22].”

“The mediation by a construct that does not increase with age may indicate the preexisting issues associated with anxious attachment, such as a low threshold for rapid and intense activation of the attachment orientation and corresponding deactivation of controlled mentalizing processes, leading to automatic mentalizing [17].”

“This aligns with the attachment literature, which indicates that individuals with anxious attachment exhibit a rapid and extensive surge of negative emotions [75].”

“This vulnerability can be seen – although tendatively in the context of the present study – in combination with the still-developing mentalizing brain areas, to suggest a high likelihood of automatic rather than controlled mentalizing, reinforcing the idea that university students could be considered at risk.”

We also removed a reference to a neurological study [24] Sebastian CL, Fontaine NMG, Bird G, Blakemore SJ, De brito SA, Mccrory EJP, et al. Neural processing associated with cognitive and affective theory of mind in adolescents and adults. Social Cognitive and Affective Neuroscience 2012; 7:53–63. https://doi.org/10.1093/scan/nsr023 PMID: 21467048 replacing with “Freda, M. F., Esposito, G., & Quaranta, T. (2015). Promoting Mentalization in Clinical Psychology at Universities: A Linguistic Analysis of Student Accounts. Europe’s Journal of Psychology. 2015;11(1): 34–49. [doi.org/10.5964/ejop.v11i1.812](https://doi.org/10.5964/ejop.v11i1.812)” that is purely psychological.

Reviewer #2: This study provides valuable insights into how emotional factors and mental health influence undergraduate students' academic experiences, with a particular focus on differences between third- and fourth-year students. The research's focus on mentalizing, emotion regulation, and psychological mindfulness provides a comprehensive framework for understanding how attachment styles influence students' emotional and cognitive processes. The large sample size (460 participants) and the clear differentiation between third- and fourth-year students contribute to the robustness of the findings. The study's suggestion that emotion regulation factors such as reappraisal and curiosity may mediate the relationship between attachment styles and epistemic trust represents an important and interesting addition to the field of educational psychology. This research could inform interventions to support students' mental health and emotional well-being, particularly in higher education institutions.

*-Comment:* However, while the study offers interesting results, there are several areas for improvement. The predominantly female sample (96%) raises concerns about the generalizability of the findings to a more diverse student population.

*Answer:* Thank you for this comment. This concern has been addressed in the Limitations section in the previous version:

“Another significant limitation is the disproportionate number of female participants, stemming from a notable imbalance in favor of women in Schools of Social Sciences from which the participants were recruited. Previous studies have shown gender differences in mentalizing and psychological mindedness among young adults [8]. The predominance of social studies students also restricts the generalizability of the study results to other academic disciplines”.

However, the clarification “to a more diverse student population” has been added in the text.

“The predominance of social studies students also restricts the generalizability of the study results to a more diverse student population, and to other academic disciplines. Using a clinical sample, such as students referred to a university counseling center, might reveal different associations among variables. Exploring secure attachment could offer a complementary perspective to the current study's findings.”

*-Comment:* Additionally, the lack of a clear explanation of how the concepts of (Psychological mindfulness) and emotional regulation were measured may make it difficult for readers to fully understand the reliability and validity of the findings.

*Answer:* The concepts of Psychological Mindedness (not mindfulness) and emotional regulation were measured with self-report instruments clearly described in the Measures section of the manuscript. Furthermore, psychometric properties of these measures have been reported in Karagiannopoulou et al. (2024) as stated in the manuscript: “Since the data were taken from a greater study, convergent and structural validity of the Greek versions of the original scales have been discussed elsewhere and have been found to have satisfactory psychometric properties [58]”.

*-Comment:* The study's inability to demonstrate a mediating role for emotional regulation factors between anxious attachment and cognitive confidence raises questions about the robustness of the proposed theoretical framework.

*Answer:* The results support previous studies and well-established findings that draw for the work of both Mikulincer & Shaver (2016) and Bateman & Fonagy (2019). The activation of the attachment system in insecure attached individuals, particularly those reporting anxious attachment, and the automatic rather than controlled mentalizing indicating the dominance of the attachment system, supports the poor mediating role of emotional regulation variables explored in the study.

*-Comment:* Furthermore, the article could benefit from a deeper exploration of how the findings could directly inform practical interventions in higher education, as the implications for teachers and counselors remain somewhat ambiguous.

*Answer:* Practical interventions in higher education aligned with the study’s findings have been added into the Discussion section

“Considering mentalizing as not just an individual but a social process, universities can create networks of relationships that facilitate thinking and appropriate action through mirroring, particularly in stressful situations, which in turn supports learning and affiliation [17;80]. Furthermore, findings suggest that interventions aimed at enhancing mentalization should be tailored developmentally, especially in early-to-mid adolescence [8]. Mentalizing can inform practical interventions in higher education by fostering university students’ reflective abilities through structured group settings, where they could explore interpersonal dynamics and emotional experiences in real time. Moreover, students through narrative writing tasks with guided reflection could enhance the development of self-other awareness and metacognitive skills [24].”

*-Comment:* Finally, the study could have included more diverse demographic variables to provide a more comprehensive view of emotional factors and mental health in higher education.

*Answer:* According to the comment, the following sentence was added into the Limitations section “Further studies could take into account more diverse demographic variables to provide a more comprehensive view of emotional factors and mental health in higher education.”

*-Comment:* Make sure that the references follow the style specified by the journal.

*Answer:* All necessary adjustments have been made so that references adhere to the journal’s specifications.

We hope that the changes we have made have addressed your concerns and improved the clarity and quality of the manuscript. We thank you again for the opportunity to revise our manuscript in a way that would fit best in the publication list of the journal.
